# Ethnicity, gender and risky sexual behaviour among Nigeria youth: an alternative explanation

**DOI:** 10.1186/s12978-017-0284-7

**Published:** 2017-01-31

**Authors:** Clifford Odimegwu, Oluwaseyi Dolapo Somefun

**Affiliations:** Demography and Population Studies, School of Public Health and Social Sciences, Faculty of Humanities, University of the Witwatersrand, Johannesburg, 2000 South Africa

**Keywords:** Youth, Condom use, Ethnicity, Multiple Partnership, Sexual debut

## Abstract

**Background:**

While studies in demography and public health have acknowledged the role of ethnic differences, the influence of ethnicity on youth sexual behaviour in Nigeria has received little or no attention. It is important to know how cultural norms and gender roles, which vary by ethnicity, may promote or prevent risky behaviour. Such information could provide insights into previously undetected sexual behaviour in multi-ethnic situations.

**Methods:**

The Nigeria Demographic and Health Surveys (NDHS) for 2003, 2008 and 2013 were pooled to examine the relationship between ethnicity and youth sexual reproductive health, proxied by age at sexual debut, multiple sexual partners (MSP) and condom use at last sexual activity, among the 6304 females and 1549 males who reported being sexually active in the four weeks preceding the survey. Multivariate analysis using a Cox proportional hazard regression model was used to determine the risk factors for early sexual activity among young people (15–24). Logistic regression was used to predict condom use at last sexual activity and MSP.

**Results:**

The median age at first sexual activity was 16 for females and 17 for males. 43% of male youths used condoms in their last sexual activity, compared to only 16% among females and a higher number of males (81%) had multiple sexual partners compared to females (35%). For females, elevated risks of first sex was higher among Hausa/Fulanis aged 15–19 and elevated risk of first sex was higher among Yoruba males.

**Conclusion:**

This study provides further evidence that in order to promote protective sexual behaviours among youth in Nigeria, social, cultural and gender-specific tactics should be put in place for the prevention of HIV and other STIs.

## Plain English Summary

Desired progress has not been made in the reduction of sexually transmitted infections and young people aged 15–24 are particularly at risk due to their high rates of risky sexual behavior. In this study, we examine the effect of ethnic origin on three measures of risky sexual behavior; age at first sex, condom use and multiple sexual partners among youth in Nigeria. We study youth in the Nigerian context because the country has a youthful population and understanding the influence of ethnicity on the sexual behavior of youth can help them make healthy transitions to adulthood.

Cross-sectional data were analyzed separately for males and females because we hypothesized that cultural characteristics of each ethnic origin that predict sexual behavior among youth would differ by sex.

Our results confirmed our hypothesis as ethnic origin stood as a significant predictor of youth sexual behavior among youth in Nigeria. We conclude that interventions that look beyond individual level should be considered in reducing the number of youth engaging in risky sexual behavior. Community based intervention programs should be developed in order to reduce risky sexual behavior among youth in Nigeria.

## Background

Young adults are at risk of negative health consequences associated with early and unsafe sexual activity. These consequences may include infection with the human immunodeficiency virus (HIV), other sexually transmitted infections (STIs) and unintended pregnancies. Nigeria, with an estimated population of 160 million [1], is second only to South Africa in the number of people living with HIV/AIDS worldwide, with 9% of the global burden of the disease being in Nigeria [1, 2]. Although efforts have been put into place by the international community and the Nigerian government to limit the spread of HIV/ADIS in the country, it still maintains an upward trajectory in certain states due to the risky sexual behaviours youth engage in. About 20,000 girls under the age of 18 give birth daily in developing countries, with Nigeria no exception. Early childbearing poses serious consequences to the health and development of young girls [3].

Risky sexual behaviour involves the number and types of partnerships, sexual acts, and sexual orientation. Other elements of risky sexual behaviour include early age at first sexual intercourse, unprotected sexual intercourse with ‘at risk’ sexual partners, and untreated sexually transmitted diseases [4]. These behaviours have implications in the prevention of HIV and other STIs. To ensure the decline of HIV and other STIs, further research on the determinants of youth sexual behaviour in Nigeria is important, especially in a country like Nigeria where 63% of the population is under 25 years old. It is critical to understand the social and cultural mechanisms underlying sexual behaviour that may be conducive to unintended pregnancies and the spread of HIV/AIDS, so as to help these youth make healthy transitions to adulthood.

There is a need to look beyond individual determinants and include sociocultural contextual factors influencing sexual behaviours of youth [5–7]. The danger of focusing on the individual psychological process alone is that it overlooks the associations of behaviour to social, cultural, and economic dimensions, thereby missing the possibility to fully recognize essential determinants of behaviour. This is because fundamental barriers that would hinder the promotion of youth protective sexual behaviours are deep-rooted within the sociocultural contexts that shape the youth [7]. Societal norms and gender-power relations influence behaviour, which may allow positive or negative changes [8]. For instance, in different societies, norms and beliefs of suitable roles for men and women are enforced by that society’s institutions and practices, such as marriage, polygamy, and female genital mutilation, among others [9–12]. This determines the extent to which men and women are able to control the various aspects of their sexual lives, i.e. their ability to negotiate the timing of sex, conditions under which it takes place, and condom usage. This plays a critical role in determining their respective vulnerabilities to HIV. For example, femininity often requires women to be passive in sexual interactions and ignorant of sexual matters, limiting their ability to access information on the risks of sex or to negotiate condom usage [13]. Masculinity, on the other hand, requires that men be sexual risk takers and condones multiple partners which, without adequate prevention, increases their vulnerability to HIV [14, 15]. The unequal power balance between men and women results in their unequal access to HIV information, resources and services.

Ethnicity is an important sociocultural factor mediating sexual behaviour in sub-Saharan Africa [16, 17] and some studies have concluded that ethnicity may be more important than socio-economic characteristics [18–20]. Ethnicity has been described a social group that shares a common and distinctive culture, religion and language. Gender, class and sexuality are the greatest sources of ethnic stability and instability, even though ethnic boundaries and identities are usually based on language and religion [21].

The relationship between ethnic origin and health outcomes has received scholarly attention [22–25]. For instance, [26] studying the effect of internal migration and premarital sexual initiation in Nigeria found ethnicity to be a key independent predictor of premarital sexual initiation. Ethnic origin *per se* does not influence health outcomes, but rather the socioeconomic characteristics of ethnic groups [27]. [26]. Ethnographic and epidemiological studies have confirmed that adolescent sexual behaviour varies from prevention to liberalism across different cultural groups [28]. Particular ethnic practices may increase the likelihood of HIV infections among young women, for instance, the practice of early marriage in some ethnic groups increases likelihood of infections and obstetric fistula [29, 30]. Ethnicity may influence sexual behaviour through cultural beliefs and practices. For example, the practice of levirate marriage, where a man’s widow is forced to remarry to one of his brothers, is still being practised in some areas of sub-Saharan Africa despite the high prevalence of HIV [31, 32].

Nigeria is home to about 374 ethnic groups and English is the official language. Three ethnic groups - Yoruba, Igbo and Hausa - make up around 50% of the population. The other much smaller minority ethnic groups, which include Kanuri, Edo, Ijaw, Ibibio, Ebira, Nupe, and Tiv, make up the rest [33]. The three major ethnic groups are differentiated not only by region, but also by religion and lifestyle. Hausa/Fulanis inhabit the northern part of Nigeria and practise the Sharia or Islamic law, and they have lower levels of educational attainment compared with ethnic groups such as the Igbos and the Yoruba. The Hausa/Fulani also have a higher proportion of illiterate adults and less access to healthcare, which may affect health behaviour [33].

Yoruba and Igbo girls tend to marry in the third decade of life, while early marriage before age 16 is common among the Hausa/Fulani [34]. This increases younger age at first birth and maternal mortality [35]. The Igbos from south-eastern Nigeria are family-oriented, possess a strong kinship system [36], and are highly patriarchal [37]. Male privilege in the form of traditional titles, land ownership, and decision-making is prevalent and women cannot own land or make decisions. This culture has been preserved for hundreds of years and passed down to younger generations [38]. Compared to the Yoruba, Hausa/Fulani and other ethnic groups, the Igbos value Western education [36], are very industrious and have a later age at marriage.

Ethnic concentration in a particular community can influence youth decision to engage in protective or risky sexual behaviour [26, 39]. However, there is not enough literature on the mechanisms of this association. Therefore understanding the extent to which ethnicity explains differences in youth sexual behaviour is important. This study specifically seeks to explore the relationship between ethnicity and youth sexual behaviour in Nigeria. It is important to study ethnicity because it serves as a proxy for different cultural norms which are otherwise hard to quantify. This paper adds to the existing literature by going beyond the normal factors associated with sexual behaviour and looking at culture.

This study hypothesizes that: 1) female Hausa/Fulani youth are more likely to have an early sexual debut compared to Yoruba and Igbo youth because of their cultural characteristics; 2) female Igbo and Yoruba youth are more likely to engage in protective sexual behaviour (condom use) as they are more likely to be educated, which may enable them understand the risk factors associated with non-condom use. We also hypothesize that the effect of ethnicity might be different for male and female youth. This study is based on the Anderson’s subcultural hypothesis [40, 41], which holds that youth sexual behaviour is shaped by subgroup expectations and norms. This argues that variations in adolescent sexual behaviour are mainly due to cultural norms and practices peculiar to particular groups. Another explanation for this hypothesis is the patriarchal system that exists in many part of Africa; males are in a position of power and authority and sanctions may be severe for females who engage in non-marital sexual behaviour [42].

The study will contribute to the body of knowledge on how ethnicity and gender differences influence higher-risk sexual behaviour among youth. The objective is to help planners and policymakers in government agencies and NGOs develop substantive, alternative policy interventions to address risky youth sexual behaviour and its consequences. Factors that influence sexual risk behaviours differ greatly between males and females and a majority of studies existing have not separated the two groups. This study aims to fill that gap.

## Methods

The data sets used in this study were the 2003, 2008 and 2013 Nigeria Demographic and Health Surveys (NDHS) [43], pooled to maximise the sample size. Asides increasing number of observations, another advantage of combining three different surveys is that it is expected that increasing the overall sample size should lead to reduced sampling errors [44]. There were no differences in sexual behaviour of male and female youth between survey years, so the analysis was not affected by sexual behaviours changing over time. A separate analysis was conducted for females and males. This is based on the premise that gender differences in norms for sexual behaviour exist and factors associated with sexual relations vary by sex. In general, males tend to have more sexual partners than females [45, 46], and they also tend to use condoms more consistently than women during vaginal intercourse [47]. The sample consisted of sexually active (i.e., “active in the last four weeks”) youth aged 15–24.

The numbers of female youth in the three cross-sectional surveys were 263, 1232 and 1489 in 2003, 2008 and 2013 respectively, giving a total of 6304 female youth. The numbers of male youth were 216, 1127 and 1359 respectively, giving a total of 1549 male youth. The survey collected information on various demographic and health indicators, including individual characteristics, marriage and sexual activity, family planning knowledge and use, and HIV/AIDS-related knowledge, attitudes and behaviour.

### Outcome variables

The sexual behaviours among youth measured in this study include early sexual debut, condom use at last sex and multiple sexual partners. Age at first sex was derived from the question “how old were you when you had sex for the first time?”. This is a continuous variable. Sexual debut at 15 years or younger was defined as early sexual debut.

Condom use was deduced from the question “Used a condom the last time had sex in the last 12 months?”. A 12-month reference period is useful for capturing the most recent behaviours and minimizes recall errors [4]. Youth were coded ‘1’ if they reported use of condom at last sexual intercourse and ‘0’ otherwise.

The number of sexual partners in the year preceding the survey was derived from the question “in the past year, how many people, if any, have you had sexual intercourse with?”. It is included in the analysis as a dichotomous variable coded ‘1’ if a man or a woman reported involvement with multiple sexual partners in the 12 months prior to the survey and ‘0’ otherwise. The focus is on the number of sexual partners because multiple and concurrent partnerships are the key mechanism through which STIs and HIV infections are spreading across sub-Saharan Africa. We consider number of sexual partners because having multiple partners is associated with disease risk for at least two reasons: first, it reflects the increased likelihood of encountering a sexually transmitted pathogen through having multiple potential exposures, and second, it may reflect an increased probability of choosing a partner with an infection through a riskier pattern of partner recruitment [48].

### Independent variables

The key independent variable in this study is ethnicity. Of the 374 identifiable ethnic groups in Nigeria, the three major ethnic groups are Hausa/Fulani, Igbo and Yoruba. They account for 60% of population, while the Edo, Ijaw, Kanuri, Ibibio, Ebira Nupe, Tiv and other minorities make up the remaining 40%. The middle belt of Nigeria is known for its diversity of ethnic groups, including the Pyem, Goemai, and Kofyar [49]. The Hausa/Fulani were categorized as one group in this study because the two tribes share a common language and common set of customs and values [50], the other minority ethnic groups were grouped as one and they were regarded as ‘others’ in this study.

Based on existing studies, we have identified some significant demographic and socioeconomic predictors of risky sexual behaviours. These variables include age, education level, employment status, religious affiliation and wealth status, which is a proxy for household socioeconomic status captured through a wealth index based on household possessions and amenities. Age may influence sexual behaviour: studies have shown that younger adolescents are at increased risk of HIV infection because they often engage in unprotected sexual intercourse [51–53]. Finer and Philbin [54] found that the low rates of contraceptive use at first sex may be due to the lower odds of having information about and access to contraceptive methods among young teens, and other scholars believe that being an older youth should be associated with protective sexual behaviours as older youth are more likely to have better knowledge and experience, which may influence their condom/contraceptive behaviour [55].

We consider religious affiliation because it has been argued that religiosity may discourage risky behaviour and therefore serve as a barrier to HIV infection. Religion can influence sexual behaviour through intermediate factors such as the age at first sex, marital status, and access to information and services.

Other variables considered include HIV knowledge. This study uses five variables to capture HIV related knowledge among men and women, these same variables have been widely used in other studies [56–58]. Similar to [59], HIV knowledge was deduced from questions such as: consistent use of condom during sexual intercourse and having just one uninfected faithful partner can reduce the chance of getting the AIDS virus, as well as having “basic knowledge” about HIV (knowing that a healthy-looking person can have the AIDS virus, and rejecting the two following most common local misconceptions about AIDS transmission or prevention: HIV transmission by mosquito bites and sharing food). Youth who responded “yes” to at least each of the questions were categorized as “High” and youth who responded “no” to all of the questions were categorized as “low”.

Socioeconomic status was considered because the association between socioeconomic status and youth risky sexual behaviour have varied [60–62]. For instance, a qualitative study in a semi-urban area in Ethiopia found that transactional sex was one of the reasons females initiated sex. This may be as a result of their low socio-economic status. According to a study using data from 26 countries, male youth whose family income fell in the middle to the highest wealth index quintile were more likely to engage in risky sexual behaviour [63]. However another study in using national surveys of adolescents in four African countries found a weak association between wealth status and sexual debut among males but wealthiest girls were more likely to have a late sexual debut [60].

The impact of exposure to mass media is investigated because media-based health promotion campaigns can substitute for formal education, by increasing understanding of health issues and/or providing knowledge of good sexual practices. The media was said to have both negative and positive influence. Exposure to television has been found in quantitative studies as a key correlate to onset of early sex and this was confirmed by another study done on Nigerian adolescents [64, 65]. Exposure to sexuality and reproductive health information and education through exposure to mass media is expected to provide sexually active individuals with the knowledge and confidence to make informed and health-oriented choices about their bodies and sexuality. Regular exposure to sexuality information can help people acquire the skills to negotiate relationships and safer sexual practices, including whether and when to engage in sexual intercourse. Accordingly, media exposure is expected to reduce the likelihood of involvement in high-risk sexual behaviour. The variable ‘media exposure’ is included as a dichotomous variable coded ‘1’ if a respondent reported that he/she watches TV or listens to radio at least once a week and ‘0’ otherwise.

Employment is measured as a dichotomous variable coded ‘1’ if a male or a female respondent was engaged in paid work and ‘0’ otherwise. The impact of cash work is difficult to predict for two reasons. On the one hand, cash work can increase propensity to engage in high-risk sex by increasing exposure to opportunities that can be used to address occasional emotional and/or economic needs. On the other hand, cash work can produce an opposite effect by making it possible for a man or a woman to develop life aspirations that make it difficult to have multiple sexual partners. The urban residence control is also crucial here because both educational opportunities and most of the components of the wealth index are highly concentrated in urban agglomerations. We used head of household as a proxy for family structure. This was categorized as “male-headed” and “female-headed”.

### Analysis

Separate analyses were performed for females and males. The descriptive statistics show the distribution of youth by the key variables. Values were expressed as absolute numbers (percentages) and median (± standard error) for categorical and continuous variables respectively. For condom use at last sex and multiple sexual partnerships, simple logistic regressions were used to obtain estimates of the odds ratios.

For age at first sex, both the descriptive and multivariate analyses were based on survival analysis techniques. Some of the individuals had not had sex as of the time of the survey therefore the variable was right-censored. We used Kaplan-Meier life tables to analyse the effects of each independent variable on the timing of first sexual experience. These estimates were nonparametric and were not subject to biases due to violations of distributional assumptions of the underlying hazard. The multivariate analysis was based on a proportional hazard model that does not assume any specific functional form for the baseline hazard. We tested for proportionality by inspecting the log survival function plotted for each category of each independent variable. The log-rank test was used to test the hypothesis of no difference in survival between the categories.

Sampling weights were applied to adjust for differences in probability of selection and to adjust for non-response in order to produce the proper representation. Individual weights were used for descriptive statistics in this study, using Stata 12 for Windows. Results on measures of association were presented as hazard ratio (HR) for age at first sex, odd ratio (OR) for condom use and multiple sexual partnerships and 95% confidence interval (CI), with alpha level set at 0.05.

### Ethical consideration

The Nigerian DHS can be downloaded from the website and is free to use by researchers for further analysis. In order to access the data from DHS MEASURE a written request was submitted to the DHS MACRO and permission was granted to use the data for this survey.

## Results

### Characteristics of respondents

#### Descriptive results

Weighted descriptive statistics for the sexually active sample are shown in Table 1. Overall, female youth reported a median age at first intercourse of 16. Males reported a statistically significantly higher age than females (17 vs 16). Regarding risky behaviour, more than half of the youth did not use a condom at last sexual intercourse, but this was higher in female youth (84%) compared to males (57%). More than a third (35%) of the female youth and about four-fifths of male youth (81%) had multiple sexual partners.

**Table 1:** Characteristics of sexually active youth

Dependent variables	Females– (*N* = 6 304)	Males– (*N* = 1,549)
Condom use at last sex		
No	83.87	57.06
Yes	16.13	42.94
Multiple sexual partners		
No	65.39	19.31
Yes	34.61	80.69
Age at first sex	Median = 16, s.d = 2.23	Median = 17, s.d = 2.49
Independent variables		
Ethnicity		
Yoruba	13.86	18.19
Igbo	11.82	11.63
Hausa/Fulani	29.42	8.00
Others	44.90	62.19
Age		
15–17	15.23	6.59
18–19	19.55	15.96
22–24	65.22	77.45
Religion		
Catholic	11.14	18.06
Other Christian	43.91	52.01
Muslim	44.16	28.07
Other	0.79	1.86
Region		
South West	15.93	21.23
North Central	14.10	10.25
North East	25.56	5.64
North West	8.48	7.92
South East	24.22	29.44
South South	11.71	25.51
Place of residence		
Urban	36.58	36.76
Rural	63.42	63.24
Schooling		
No education	30.49	6.59
Primary education	15.02	12.76
Secondary and higher	54.50	80.65
Employment status		
Unemployed	51.32	33.90
Employed	48.68	66.10
Wealth status		
Poor	35.53	27.02
Middle	19.67	22.48
Rich	44.80	50.50
HIV knowledge		
Low	3.46	0.85
High	96.54	99.15
Marital status		
Never married	33.28	77.79
Married	61.92	18.81
Other	4.80	3.40
Exposure to mass media		
No	58.53	36.04
Yes	41.47	63.96
Head of household		
Male	83.35	85.76
Female	16.65	14.24
Self-reported age at sexual debut		
10–19 Adolescents	90.53	83.73
20–24 Young Adults	9.47	16.27

Fifty-six percent of the female youth belong to the three major ethnic groups (Hausa, Igbo, and Yoruba), while 48% of the male youth belong to these groups.

Female and male youth aged 22–24 constitute more than two-thirds of the total sample. One reason for this could be that there may have been a selection bias where younger participants were not approached or declined to take part in the survey. About half of the adolescents were Christians (44% of females and 52% of males) with less than one quarter were Catholics (14% for females vs 18% for males).

More than half of the female and male youth lived in rural areas (63%). While the majority of youth have attained secondary and higher levels of education, the level of educational attainment varied by sex, that is more males than females have a secondary education (81% vs 55% respectively). More females were unemployed compared to males (51% vs 34% respectively). A higher number of male youth (64%) were exposed to mass media compared to female youth (42%).

#### Bivariate association

The distribution of risky sexual behaviour by ethnicity is presented in Table 2. Among the ethnic groups, the percentage of female youth who used a condom at last sex was lowest among the Hausa/Fulanis (1%) followed by youth in the ‘others’ category (17%). Igbo female youth were most likely to engage in multiple sexual partnerships (54%) compared to the Hausa/Fulani youth (2%). Similarly, condom use at last sex was lowest among the male Hausa/Fulani youth, at one fifth (20%). Male Igbo and Yoruba youth reported highest number of multiple sexual partners (87%) while Hausa/Fulani reported lowest (35%) among the ethnic groups. These associations were statistically significant.

**Table 2:** Percentage distribution of risky sexual behaviour by ethnicity

Ethnicity	Condom use at last sex		Multiple sexual partners	
	Females	Males	Females	Males
Yoruba	30.08	58.34	51.48	87.36
Igbo	33.31	57.13	54.28	87.43
Hausa/Fulani	0.97	20.26	1.60	35.22
Others	17.26	38.70	45.91	83.29

**Fig. 1:**
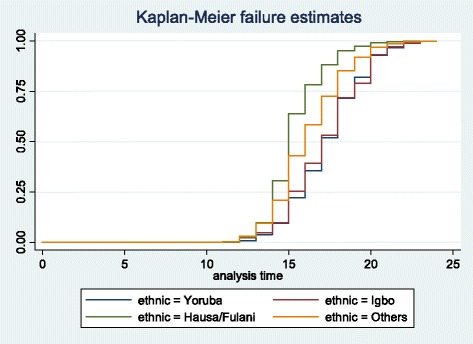
Kaplan-Meier failure estimates of ethnicity by age at first sex in Nigeria (females)

**Fig. 2:**
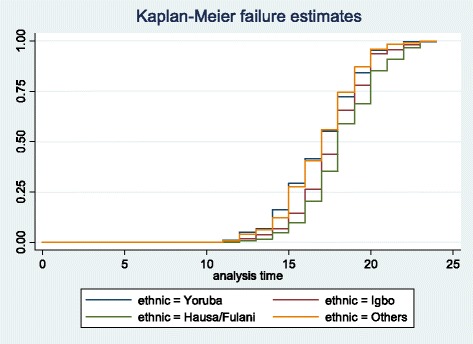
Kaplan-Meier failure estimates of ethnicity by age at first sex in Nigeria (males)

The event history analysis showing association between ethnicity and age at first sex is presented in Table 3 and the Kaplan-Meier plots are shown in Figs. 1 and 2. For females, elevated risks of first sex was higher among Hausa/Fulanis aged 15–19, but at ages 20–22, there was no difference in the risk of first sex among the ethnic groups. Results differed for male youth. Elevated risk of first sex was higher among Yoruba males and males of the ethnic groups in the ‘others’ category.

**Table 3:** Event history analysis of age at first sex by ethnicity

	Females				Males			
	Yoruba	Igbo	Hausa	Others	Yoruba	Igbo	Hausa	Others
Time	Failure Function	Failure Function	Failure Function	Failure Function	Failure Function	Failure Function	Failure Function	Failure Function
15	0.2603	0.2834	0.6453	0.4472	0.2925	0.1437	0.0984	0.2753
16	0.4104	0.4349	0.7881	0.604	0.415	0.2625	0.2049	0.4059
17	0.5896	0.5775	0.887	0.7456	0.5534	0.4375	0.3525	0.5597
18	0.7672	0.7701	0.9545	0.8678	0.7233	0.6562	0.5902	0.7449
19	0.8668	0.8342	0.9781	0.9287	0.8419	0.7813	0.6885	0.8705
20	0.9479	0.9447	0.9933	0.9737	0.9526	0.9375	0.8525	0.9585
21	0.9755	0.975	0.9983	0.9875	0.9842	0.9563	0.9098	0.9838
22	0.0031	0.9893	0.9994	0.9962	0.996	0.9812	0.9672	0.9919

*P*-value (log rank test) – 0.000

#### Multivariate analysis

To examine the effects of gender on the timing of first intercourse, we conducted two multivariate analyses using proportional hazard techniques. In the first, we estimated the gross effects of ethnicity; in the second, we estimated the net effects of ethnicity controlling for other covariates in Table 4. Ethnicity was seen to be significantly associated with age at first sexual debut for both female and male youth. Compared with Yoruba youth, Hausa/Fulani females had a significantly higher rate of early first sex (HR 2.18, C.I. 2.01–2.36). On the contrary, Hausa/Fulani males had significantly lower hazards of first sex (HR 0.63, C.I. 0.52–0.77) and a lower hazard of early sexual activity was also seen amongst Igbo males (HR 0.76, C.I. 0.63–0.92) compared to Yoruba male youth. These associations were statistically significant.

**Table 4:** Adjusted and unadjusted coefficients of the association between ethnicity and age at first sex

	Females		Males	
	Unadjusted	Adjusted	Unadjusted	Adjusted
Ethnicity				
Yoruba				
Igbo	1.00 (0.92–1.09)	0.89 (0.77–1.04)	0.76*** (0.63–0.92)	0.71** (0.53–0.95)
Hausa/Fulani	2.18*** (2.01–2.36)	1.11 (0.96–1.28)	0.63*** (0.52–0.77)	0.94 (0.67–1.31)
Others	1.39*** (1.30–1.49)	1.02 (0.90–1.15)	0.97 (0.85–1.12)	1.03 (0.79–1.33)
Religion				
Catholic				
Other Christian	0.99 (0.92–1.07)	0.95 (0.87–1.03)	0.96 (0.83–1.12)	0.94 (0.81–1.10)
Muslim	1.55*** (1.43–1.67)	1.06 (0.95–1.18)	0.79*** (0.67–0.93)	0.87 (0.73–1.04)
Other	1.36*** (1.06–1.74)	0.85 (0.64–1.12)	0.95 (0.61–1.48)	0.92 (0.59–1.45)
Region				
South West				
North Central	1.85*** (1.70–2.02)	1.07 (0.93–1.23)	0.74*** (0.62–0.89)	0.70* (0.53–0.93)
North East	2.05*** (1.89–2.23)	1.17** (1.02–1.35)	0.59*** (0.47–0.73)	0.59*** (0.43–0.81)
North West	1.00 (0.91–1.09)	1.10 (0.93–1.30)	0.81* (0.65–1.00)	1.01 (0.72–1.42)
South East	1.31*** (1.22–1.42)	1.30*** (1.14–1.47)	1.00 (0.86–1.16)	0.90 (0.69–1.170
South South	1.27*** (1.17–1.38)	0.95 (0.84–1.02)	0.99 (0.85–1.15)	0.88 (0.68–1.13)
Place of residence				
Urban				
Rural	1.56*** (1.49–1.64)	1.13*** (1.06–1.21)	1.09 (0.99–1.21)	1.04 (0.92–1.17)
Schooling				
No education				
Primary education	0.75*** (0.69–0.82)	0.93 (0.85–1.03)	1.73*** (1.36–2.18)	1.50*** (1.15–1.96)
Secondary and higher	0.45*** (0.43–0.48)	0.67*** (0.61–0.74)	1.48*** (1.21–1.81)	1.34*** (1.04–1.73)
Employment status				
Unemployed				
Employed	0.97 (0.92–1.01)	1.00 (0.95–1.05)	0.96 (0.87–1.06)	1.02 (0.91–1.13)
Wealth status				
Poor				
Middle	0.70*** (0.65–0.75)	0.84*** (0.78–0.91)	1.03 (0.89–1.20)	0.90 (0.77–1.06)
Rich	0.50*** (0.47–0.53)	0.75*** (0.69–0.82)	0.93 (0.82–1.05)	0.83 * (0.70–0.97)
HIV knowledge				
Low				
High	0.82** (0.71–0.95)	1.02 (0.88–1.18)	1.30 (0.41–4.13)	1.11 (0.39–3.20)
Exposure to mass media				
No				
Yes	0.71*** (0.68–0.75)	0.91*** (0.86–0.97)	0.99 (0.88–1.10_	0.94 (0.84–1.06)
Head of household				
Male				
Female	0.81*** (0.76–0.86)	0.96 (0.90–1.02)	1.25*** (1.10–1.42)	1.16* (1.01–1.34)

* = *p* < 0.1 (significant at 10%)

** = *p* <0 .05 (significant at 5%)

*** = *p* < 0.01 (significant at 1%)

Religion, region, educational level, and family structure were associated with age at sexual debut for both female and male youth at the bivariate level. In addition, place of residence, wealth status; HIV knowledge and exposure to mass media were associated with early sexual debut for female youth but not for males.

Controlling for other covariates in the adjusted model, ethnicity significantly predicted age at first sex male youth only, as there was no association between ethnicity and sexual debut of female youth. Results showed that Igbo males had significantly lower hazards of early sexual activity (HR 0.71, C.I. 0.53–0.95) and the effects of Hausa/Fulani males were reduced to non-significance.

In the adjusted model, there was no association between religion and age at sexual debut. Region and educational level remained significant predictors among female and male youth. Place of residence and wealth status were factors predicting age at sexual debut among females. Among males, wealth status became a significant predictor of age at sexual debut.

#### Demographic factors

For example, being a female from a rural area (HR 1.13, C.I. 1.06–1.21) was significantly associated with higher rates of early sexual activity and being exposed to mass media (HR 0.91, C.I. 0.86–0.97) was significantly associated with lower rates of early sexual activity among female youth.

#### Socio-economic factors

Having secondary and higher education (HR 0.67, C.I. 0.61–0.74) and belonging to a rich household (HR 0.75, C.I. 0.69–0.82) was significantly associated with lower rates of early sexual activity among female youth.

On the other hand, for male youth, having secondary and higher education (HR 1.34, C.I. 1.04–1.73) and being a member of a female-headed household (HR 1.16, C.I. 1.01–1.34) were associated with elevated risk of early sexual debut.

Table 5 shows results of the odds ratios, showing the effects of ethnicity on condom use at last sex and multiple sexual partnerships for females and males, controlling for sociodemographic and other characteristics. The result confirms the proposition that there is ethnic variation in youth sexual behaviour among adolescents in Nigeria.

**Table 5:** Multivariate regression models predicting odds of risky sexual behaviours among youth

	Females				Males			
	Condom Use Last Sex		Multiple sexual partners		Condom Use Last Sex		Multiple sexual partners	
	Unadjusted	Adjusted	Unadjusted	Adjusted	Unadjusted	Adjusted	Unadjusted	Adjusted
Ethnicity								
Yoruba								
Igbo	1.16 (0.89–1.49)	1.31 (0.70–2.44)	1.25 (0.96–1.63)	0.75 (0.43–1.32)	0.95 (0.60–1.49)	0.82 (0.35–1.88)	1.0 (0.49–2.03)	0.29 (0.05–1.49)
Hausa/Fulani	0.02*** (0.01–0.03)	0.49 (0.21–1.12)	0.01***(0.01–0.02)	0.07*** (0.03–0.15)	0.18*** (0.10–0.32)	1.39 (0.54–3.55)	0.07 *** (0.04–0.14)	0.65 (0.14–2.93)
Others	0.48*** (0.39–0.59)	0.96 (0.60–1.53)	0.93 (0.76–1.13)	0.81 (0.55–1.20)	0.45*** (0.32–0.62)	0.82 (0.43–1.56)	0.72 (0.42–1.20)	1.08 (0.26–4.37)
Age								
15–17								
18–19	1.81*** (1.37–2.39)	1.58*** (1.15–2.19)	1.09 (0.89–1.32)	0.40*** (0.29–0.54)	1.15 (0.67–1.99)	0.93 (0.51–1.71)	1.13 (0.38–3.33)	5.43*** (1.56–18.92)
22–24	1.56*** (1.22–1.99)	1.44** (1.06–1.96)	0.51*** (0.43–0.61)	0.11*** (0.09–0.15)	1.32 (0.82–2.11)	1.27 (0.74–2.20)	0.29*** (0.13–0.64)	3.32** (1.15–9.55)
Religion								
Catholic								
Other Christian	0.90 (0.72–1.12)	0.91 (0.67–1.24)	1.16 (0.95–1.43)	0.86 (0.65–1.14)	1.1 (0.72–1.42)	0.86 (0.56–1.30)	1.33 (0.81–2.19)	1.07 (0.51–2.24)
Muslim	0.13*** (0.10–0.17)	0.69 (0.45–1.06)	0.10*** (0.07–0.12)	0.51*** (0.35–0.73)	0.70 (0.48–1.01)	0.78 (0.48–1.29)	0.41*** (0.25–0.67)	0.78 (0.26–2.29)
Other	0.33** (0.13–0.83)	2.15 (0.66–6.96)	0.16*** (0.07–0.35)	0.56 (0.23–1.33)	0.38 (0.12–1.16)	0.43 (0.15–1.26)	0.46 (0.13–1.61)	0.81 (0.17–3.75)
Region								
South West								
North Central	0.09*** (0.06–0.12)	0.64 (0.35–1.18)	0.13*** (0.10–0.17)	0.59** (0.37–0.94)	0.19*** (0.12–0.30)	0.37* (0.17–0.78)	0.30*** (0.18–0.51)	0.54 (0.16–1.85)
North East	0.07*** (0.05–0.11)	0.57 (0.28–1.16)	0.06*** (0.04–0.09)	0.67 (0.37–1.21)	0.26*** (0.13–0.52)	0.72 (0.23–2.16)	0.14*** (0.07–0.27)	0.41 (0.11–1.56)
North West	1.03 (0.78–1.37)	0.82 (0.42–1.56)	1.12 (0.84–1.48)	0.91 (0.50–1.67)	0.97 (0.60–1.57)	0.96 (0.38–2.37)	0.95 (0.47–1.90)	1.64 (0.39–6.77)
South East	0.72*** (0.59–0.90)	0.66* (0.41–1.06)	2.03*** (1.65–2.50)	1.69*** (1.14–2.50)	0.63** (0.44–0.88)	0.69 (0.3501.34)	1.57 (0.93–2.66)	0.57 (0.17–1.86)
South South	0.31*** (0.23–0.41)	0.66 (0.41–1.08)	0.50*** (0.39–0.64)	0.77 (0.52–1.14)	0.54*** (0.38–0.77)	0.90 (0.48–1.72)	0.68 (0.41–1.13)	0.78 (0.24–2.55)
Place of residence								
Urban								
Rural	0.27*** (0.23–0.32)	0.68*** (0.54–0.86)	0.49*** (0.42–0.56)	0.76** (0.61–0.94)	0.39*** (0.30–0.50)	0.72* (0.53–0.99)	0.57*** (0.41–0.79)	1.43 (0.68–3.00)
Schooling								
No education								
Primary education	6.29*** (3.75–10.54	1.30 (0.72–2.33)	10.37*** (7.31–17.71	2.70*** (1.76–4.13)	6.65***(2.39–18.49)	1.74 (0.58–5.18)	8.06*** (4.39–14.80)	2.96* (1.04–8.38)
Secondary and higher	36.84*** (23.67–57.34)	2.77*** (1.61–4.74)	45.74*** (33.38–62.66)	8.77*** (5.81–13.22)	18.98*** (7.33–49.11)	2.87* (1.02–8.07)	19.92*** (12.08–32.86)	2.77 (0.98–7.86)
Employment status								
Unemployed								
Employed	1.06 (0.90–1.26)	1.28** (1.03–1.58)	0.77*** (0.67–0.87)	0.68 (0.57–0.82)	0.62*** (0.49–0.80)	1.13 (0.84–1.52)	0.10*** (0.06–0.17)	0.47 (0.22–1.03)
Wealth status								
Poor								
Middle	2.92*** (2.19–3.88)	1.20 (0.86–1.68)	2.58*** (2.15–3.10)	1.02 (0.77–1.34)	2.53*** (1.75–3.66)	1.75***(1.16–2.63)	2.07*** (1.36–3.12)	0.63 (0.29–1.34)
Rich	7.05*** (5.54–8.98)	1.70*** (1.23–2.35)	4.43*** (3.78–5.19)	1.01 (0.76–1.33)	5.00*** (3.64–6.87)	2.45***(1.63–3.69)	3.47*** (2.46–4.91)	0.82 (0.36–1.83)
HIV knowledge								
Low								
High	2.12** (1.16–3.89)	0.86 (0.49–1.51)	3.08*** (1.94–4.86)	2.38*** (1.38–4.03)	3.69 (0.57–23.94)	1.35 (0.16–11.36)	4.71** (1.28–17.39)	1.10 (0.23–5.31)
Marital status								
Never married								
Married	0.04*** (0.03–0.06)	0.15*** (0.08–0.29)			0.09*** (0.05–0.14)	0.22***(0.11–0.44)		
Other	0.2*** (0.13–0.30)	0.29*** (0.15–0.58)			0.39** (0.19–0.80)	0.58 (0.25–1.36)		
Exposure to mass media								
No								
Yes	2.24*** (1.88–2.66)	1.17 (0.94–1.45)	1.86*** (1.63–2.12)	1.06 (0.88–1.29)	1.94*** (1.51–2.50)	1.58***(1.18–2.13)	1.66*** (1.22–2.25)	1.19 (0.65–2.17)
Head of household								
Male								
		0.97 (0.77–1.22)	7.66*** (6.38–9.18)	4.51*** (3.58–5.68)	1.30 (0.94–1.80)	1.03 (0.71–1.49)	7.46*** (3.57–15.56)	3.23** (1.23–8.44)
Female	2.97*** (2.44–3.60)							
Self–reported age at sexual								
debut								
10–19 Adolescents								
20–24 Young Adults	2.17*** (1.66–2.83)		1.63*** (1.30–2.05)		1.04 (0.76–1.43)		0.47*** (0.32–0.68)	
Multiple sexual partners								
No								
Yes	16.11*** (12.92–20.09)	7.9***6(6.11–10.38)			9.36*** (6.00–14.61)	2.73***(1.43–5.20)		

*=*p* < 0.1 (significant at 10%)

** = *p* <0 .05 (significant at 5%)

*** = *p* < 0.01 (significant at 1%)

For both genders, ethnicity was associated with condom use at last sex at the bivariate level. Hausa/Fulani females (OR 0.02, C.I. 0.01–0.03) and females of ‘other’ ethnic groups (OR 0.48, C.I. 0.39–0.59) had lower odds of using condom at last sexual intercourse compared with Yoruba. Similar results were seen for the males. Hausa/Fulani males (OR 0.18, C.I. 0.10–0.32) and males of ‘other’ ethnic groups (OR 0.45, C.I 0.32–0.62) had lower odds of using a condom at last sexual intercourse compared with Yoruba youth.

For multiple sexual partnerships, similar results were seen for female and male youth. Hausa/Fulani females (OR 0.01, C.I 0.01–0.02) and males (OR 0.07, C.I 0.04–0.14) had significantly lower odds of having multiple sexual partners compared to Yoruba female and male youth.

After controlling for covariates, there was no association between ethnicity and condom use at last sex for female and male youth. For multiple sexual partnerships, Hausa/Fulani females (OR 0.07, C.I. 0.03–0.15) had significantly lower odds of having multiple sexual partners compared to Yoruba female youth. There was no significant association between ethnicity and multiple sexual partnerships for male youth. Age at sexual debut was correlated with respondent age and was dropped in the full model.

Among the other characteristics, sociodemographic factors such as region, place of residence, educational level, wealth status, marital status and multiple sexual partnerships were significantly associated with condom use at last sex for both female and male youth. Age and employment status were associated with condom use at last sex for females, but not for males, while exposure to mass media was associated with condom use at last sex for male youth and not females.

#### Demographic factors

Older female youth had increased odds of using condoms at last sex. Females aged 22–24 had 44% higher odds (OR 1.44, C.I. 1.06–1.96) of using condoms at last sex compared to those aged 15–17. Female (OR 0.68, C.I. 0.54–0.86) and male (OR 0.72, C.I 0.53–0.99) youth residing in rural areas had significantly lower odds of condom use at last sex compared to urban youth while controlling for other covariates.

Married female (OR 0.15, C.I. 0.08–0.29) and male youth (OR 0.22, C.I. 0.11–0.44) were less likely to use condoms at last sex compared to those who were unmarried. Male youth who were exposed to mass media (OR 1.58, C.I. 1.18–2.13) were about two times more likely to use condoms at last sex compared to their counterparts. Having multiple sexual partners showed increased odds in use of condoms among female (OR 7.96, C.I. 6.11–10.38) and male (OR 2.73, C.I 1.43–5.20) youth.

#### Socio-economic factors

In terms of education, female (OR 2.77, C.I. 1.61–4.74) and male (OR 2.87, C.I. 1.02–8.07) youth who had attained secondary and higher education were about three times more likely to use condoms at last sex compared to those with no education. Female youth who were working also had higher odds (OR 1.28, C.I. 1.03–1.58) of using a condom at last sex compared to those who were not working. Female (OR 1.70, C.I. 1.23–2.35) and male (OR 2.45, C.I. 1.63–3.69) youth from rich households were two times more likely to use condoms compared to their counterparts from poor households.

For multiple sexual partners, the covariates of age, educational level and family structure were significant predictors for females and males. Religion, region, place of residence and HIV knowledge were significantly associated with multiple sexual partners among females.

#### Demographic factors

Females aged 22–24 (OR 0.11, C.I. 0.09–0.15) were less likely to have multiple sexual partners compared to their counterparts aged 15–17, but males aged 22–24 (OR 3.32, C.I. 1.15–9.55) were more likely to have multiple sexual partners compared to those aged 15–17. Compared to Catholic female youth, Muslim youth had lower odds (OR 0.51, C.I. 0.35–0.73) of having multiple sexual partners. Also, youth in rural areas (OR 0.76, C.I. 0.61–0.94) had lower odds of having multiple sexual partners. Females with high HIV knowledge (OR 2.38, C.I. 1.38–4.03) were about two times more likely to have multiple sexual partners compared to their counterparts with low HIV knowledge. Female (OR 4.51, C.I. 3.58–5.68) and male (OR 3.23, C.I. 1.23–8.44) youth from female-headed households were about three times more likely to have multiple sexual partners compared to those from male-headed households.

## Discussion

In this paper, we have used three rounds of Nigerian DHS data for the period 2003–2013 to identify the associations between ethnicity and youth sexual behaviour.

The median age at first sex for females (16 years) and males (17 years) in our study is comparable to evidence from the Demographic and Health Surveys and the AIDS Indicators Surveys [66] which show that mean age at first sex among 20–24 year old women ranges from a low of 16 years or younger in Chad, Mali and Mozambique to a high of 19.6 in Senegal, while it ranged from 16.9 in Mozambique to 19.6 in Ghana among young men of the same age group [67]. Another report based on a study among adolescents 12–19 years old in four African countries, Burkina Faso, Ghana, Malawi, and Uganda, also found that adolescent females in Sub-Saharan Africa tend to have sex at an earlier age compared to their male counterparts [68]. Based on the suggestion that young men aged 15–24 are more likely to exert their masculinity by engaging in early sexual debut, we did not expect our results to indicate that males had a later age at sexual debut compared to females. One possible reason for this finding that is young women in Nigeria may assume that engaging in early sexual activity asserts their femininity [69]. In addition, it could be also be as a result of child sexual molestation of girls [70], sexual violence against females [71–73] and the patriarchal culture that encourages child brides [74].

Our results support the normative context theory that influences youth sexual behaviour. Interventions aimed at promoting abstinence should address variations in educational levels and access to reproductive health services across various ethnic groups, for example, policy makers must provide females belonging to the Hausa/Fulani ethnic group adequate information about their bodies and reproductive processes. The media would also play a role in positively educating the females in this ethnic group and more emphasis should be paid to the quality of sexual education give to the youth in this category.

In our study, which is consistent with other studies [67, 75–77]; women are less likely than men to engage in multiple sexual partnerships. Among young men who were sexually active, more than 80% of them had had multiple partners in the past 12 months, compared with fewer than 40% of young women. This may be as a result of the culturally defined gender roles that differentiate female sexuality from that of males. These findings support the subcultural hypothesis [42] which believes that males are in a position of power and authority and sanctions against female non-marital sexual behaviours are severe. These gender norms that men’s sexual desires are ‘irrepressible’ play a significant role in encouraging multiple sexual partnerships. To address and reduce the effect of gender norms, it is important that new mechanisms be developed that are able to reach young girls in rural areas. In addition, these programs must target and educate men on gender relations and masculinities. These programs could involve advocacy, educational campaigns and social reforms.

As expected, and consistent with other studies, condom use at last sex was higher for male youth compared to females [78–81]. The patriarchal relationship, which could be seen as a social norm in Nigeria, could be responsible for the association. This could be due to the fact that condoms are a male-determined method as it is usually the man who determines whether or not a condom is used and when and this could explain why males are more likely to report use than females. Also, young females may be having sex with older men and women’s difficulties in negotiating male condom use with partners remain a barrier to successful use of condoms [82].

As hypothesized, our descriptive bivariate findings suggest the possible effect of ethnicity in predicting sexual behaviour. The likelihood that female youths would engage in risky sexual behaviour is related to their ethnic background. Yoruba and Igbo male youth were more likely to use condoms at last sex and engage in multiple sexual partnerships. Hausa/Fulani female youth had an elevated risk of early sexual debut, which may be as a result of some of their socioeconomic characteristics (Appendix 1). This could be as a result of early marriages among women of these ethnic groups. Findings from the logistic regression confirm ethnicity to be a determinant of youth sexual behaviour. Hausa/Fulani youth (females and males) were less likely to use condoms at last sex, although this association became insignificant after controlling for other covariates.

This study has identified significant variations in the sexual behaviour of youth in Nigeria within ethnic boundaries. Our results reflect the suggestion that social and cultural contexts are primary determinants of youth sexual behaviour. The perceived ethnic effect is in line with the theoretical and empirical results of Knodel and Van de Walle [83] who explained the changes in the demographic transition with the cultural diffusion theory, and the work of Mberu [84] on condom use at the onset of premarital sexual relationship among youths in Nigeria. Opposition to sex education and condom use campaigns varies along ethno-religious lines, underscoring the need for targeted intervention strategies for different groups.

## Conclusion

Our findings are in support of the literature on the ecological argument that health behaviours are shaped and determined by societal conditions, even after controlling for individual and household characteristics [48]. It has also revealed that ethnicity is not sui generis; it’s also shaped by the political and economic systems around it. This can be seen in the context of the educational discrimination faced by females belonging to the Hausa/Fulani ethnic group. Also, a higher prevalence of male youth engaging in risky sexual behaviour is more likely to be seen in settings where sexual norms are liberal and where polygyny is common. In some Nigerian settings, some men, for instance, the Igbo men are raised to believe in their own superiority over women. Women are treated like property and a man has this notion that he can own as many of them as possible. The culture does not also restrict the men to sexual purity, so they will tend to have more than one sexual partner. This study has shown that ethnic origin is an important of youth sexual behaviour in Nigeria and community based interventions are important in the reduction of risky sexual behaviour among youth.

### Strength and limitations

This study is based on nationally representative household surveys that reflect every locality in Nigeria. Also, data were pooled together to create large sample sizes of youth sexual behaviour. There are some limitations, however, that need to be highlighted. The limitations include possible bias in reporting of sexual behaviours, exaggeration and under-reporting of sexual activity and number of sexual partners. In general, men tend to overstate their sexual behaviour while women tend to understate theirs due to sociocultural perceptions [85]. Furthermore, the recall of age at sexual debut, especially for older youth, might have contributed to some reporting bias. It should also be noted that due to the snapshot nature of cross-sectional studies, we cannot draw causal inferences from the findings of this study. Similarly, we cannot assert these findings as globally generalizable, because being in sexual relationships with multiple partners among youth has been found in other areas to be associated with inconsistent condom use.

## Abbreviations

AIDS: Acquired Immunodeficiency Syndrome
DHS: Demographic and Health Surveys
HIV: Human Immunodeficiency Virus
MSP: Multiple Sexual Partners
NDHS: Nigeria Demographic and Health Survey
OR: Odds Ratios
STIs: Sexually Transmitted Infections
